# Meta-analysis of the clinicopathological significance of miRNA-145 in breast cancer

**DOI:** 10.1042/BSR20193974

**Published:** 2020-09-18

**Authors:** Peng Lv, Zhenzhu Zhang, Li Hou, Yayue Zhang, Lingeng Lu, Chong Wang, Fengqin Shi

**Affiliations:** 1 Department of Oncology and Hematology, Dongzhimen Hospital Affiliated to Beijing University of Chinese Medicine, 5 Haiyuncang Street, Dongcheng District, Beijing 100700, China; 2 Cancer center, The Central Hospital of Enshi Tujia and Miao Autonomous Prefecture, No. 158 Wuyang Avenue, Enshi City, Hubei Province 445000, China; 3 Department of Chronic Disease Epidemiology, Yale School of Public Health, School of Medicine, Yale University, New Haven, CT 06520, U.S.A.

**Keywords:** breast carcinoma, MicroRNA, miR-145, meta- analysis

## Abstract

Low expression of tumor suppressor microRNA (miRNA) and high expression of carcinogenic miRNA promote the occurrence and progression of human cancer. Most studies show that miR-145 is a tumor suppressor miRNA, and is closely related to the clinicopathology of breast cancer. However, the results are still inconsistent. Therefore, we conducted a meta-analysis on the basis of eligible studies to summarize the possible correlation between miR-145 and the clinicopathology and prognosis of breast cancer. Using PubMed, Embase, Web of Science, Wanfang and CNKI, we searched all published papers written in either English or Chinese on miR-145 expression in breast cancer from 1990 to November 2019 for meta-analysis. We used standardized mean difference (SMD) to evaluate the differential expression of miR-145 in breast cancer tissues and adjacent normal tissues or normal breast tissues. We found that miR-145 expression was significantly lower in breast cancer tissues than that in adjacent normal tissues (SMD = −2.93, *P*<0.0001) and in healthy women (SMD = −0.52, *P*=0.009). miR-145 expression was lower in breast cancer patients with ER-positive (SMD = 0.65, *P*<0.001), HER-2-positive (SMD = −1.04, *P*<0.001), compared with their counterparts, respectively. In addition, breast cancer patients with low expression of miR-145 had larger tumor diameters (SMD = −1.97, *P*<0.001) and lymph node metastasis (SMD = −1.75, *P*<0.001) that are unfavorable prognostic factors. Conclusion: Low miR-145 is observed in breast cancer, which is closely related to molecular subtypes and unfavorable factors of breast cancer. These findings indicate that miR-145 is tumor suppressor miRNA, and may be a potential diagnostic and prognostic marker in breast cancer.

## Introduction

Human tumor is characteristic of gain-of-function of oncogene and/or loss-of-function of tumor suppressor genes. MicroRNAs (miRNAs) are a class of small non-coding RNAs of approximately 21–24 nucleotides in length that post-transcriptionally regulate the expression of its target genes [1]. MiRNAs induce degradation or translation inhibition of target genes by partially or completely pairing with bases in the 3′-untranslated region [2]. Each miRNA has numerous targets, and plays a vital role in cell development, proliferation, differentiation, chromatin structure, apoptosis, metabolism and morphogenesis [3]. The aberrant expression of miRNA frequently occurs in human cancer including breast cancer, and miRNAs are a potential diagnostic and prognostic markers and therapeutic targets.

In China, breast carcinoma accounts for approximately 15% of the total number of newly diagnosed cancer in women, and the incidence of breast carcinoma ranks the first among all cancers, among women [4]. Breast carcinoma mortality exhibits an increasing trend and is the leading cause of deaths among women with cancer [4]. Thus, it is a priority to seek the molecular targets for early diagnosis, prognosis and development of new targeted drug in breast cancer with the focus on miRNAs in the past decade.

Among hundreds of miRNA molecules, the relationship between miR-145 and breast cancer has attracted attention. Yang et al. showed that miR-145 acted as a tumor suppressor miRNA and its expression decreased in breast cancer tissues. Restoring miR-145 could inhibit the proliferation of breast cancer cells by down-regulating *HBXIP* gene [5]. Zou et al. showed that the expression of miR-145 in breast cancer tissues was significantly lower than that in adjacent tissues and normal breast tissues, and it was confirmed in *in vitro* cell experiments that miR-145 inhibited tumor angiogenesis and growth through suppressing N-RAS and VEGF, thereby playing a role in tumor suppression [6]. However, some other studies have also shown that there is no difference in the expression of miR-145 in breast normal tissues and breast cancer tissues with different clinical stages [7,8]. These observations suggest that the results are inconsistent on the relationship between miRNAs and breast cancer. Therefore, the clinicopathological significance of miR-145 in breast cancer tissues remains controversial. Thus, in the present study, we performed systemic meta-analysis of miR-145 expression in breast cancer.

## Materials and methods

### Search strategy

We searched all published papers written in either English or Chinese on miR-145 expression in breast cancer from 1990 to November 2019. Using PubMed, Embase, Web of Science, Wanfang and CNKI, and we used the combination of keywords including ‘breast neoplasm’, ‘breast tumor’, ‘breast cancer’, ‘mammary cancer’, ‘malignant neoplasm of breast’ or ‘breast malignant neoplasm ’, ‘malignant tumor of breast’, ‘breast malignant tumor’, ‘cancer of breast’, ‘human mammary carcinoma’, ‘human mammary neoplasm’, ‘breast carcinoma’ and ‘miRNA-145’, ‘miR-145’, ‘hsa-mir-145’, ‘microRNA-145’.

### Including and excluding criteria

The criteria for papers included in the analysis are the data available for average and standard deviation of miR-145 expression in breast cancer tissue and adjacent tissues or normal breast tissue as well as by the subgroup of clinicopathological variables. If a paper did not have average and standard deviation data for analysis, we first contacted the author and tried to get them. If the author did not reply or still failed to get the mean and standard deviation data, the article was excluded. Studies that measured miR-145 expression levels in blood samples/human cell lines only, or studies that just mentioned the molecular role of target miR-145 without the expression of miR-145 in breast cancer tissues were also excluded. According to the literature quality grading method recommended by the GRADE system [9], low-quality literature were excluded. For duplicate articles, articles with the latest and largest data were included in the analysis.

### Data extraction

The data were extracted by two authors (Peng Lv and Fengqin Shi) independently from eligible articles, and any discrepancy was resolved by consensus. The first author’s name, publication year, country, sample type and size, quantitative methods and publication language of the article included in this analysis were collected.

### Statistical analysis

We applied Stata14.0 software for heterogeneity analysis of the included literature. When I-squared was greater than 50% or *P*<0.05, there was heterogeneity in the literature [10], and the Dersimonian–Laird (D–L) random-effects model was used to analyze the included data [11]. Otherwise, the Mantel–Haenszel (M–H) fixed-effects model was applied [12]. Sensitivity analysis was used to explore the source of heterogeneity, and the literature that might be the source of heterogeneity was read again and analyzed to identify the characteristics that might lead to heterogeneity. The characteristic factors of the heterogeneous literature were analyzed by the meta-regression analysis method, and then the subgroup analysis was performed based on the characteristics of the heterogeneity. For continuous data, we considered that there are some differences in the measurement instruments between the studies. Therefore, we conservatively used standardized mean difference (SMD) and 95% confidence intervals (95% CIs) to evaluate the differential expression of miR-145 in breast cancer tissues and adjacent normal tissues or normal breast tissues. At the same time, the Egger’s test and Begg’s test were used to determine publication bias. Data input and monitoring were done by Peng Lv and Fengqin Shi.

## Results

### Study characteristics

**Figure 1:**
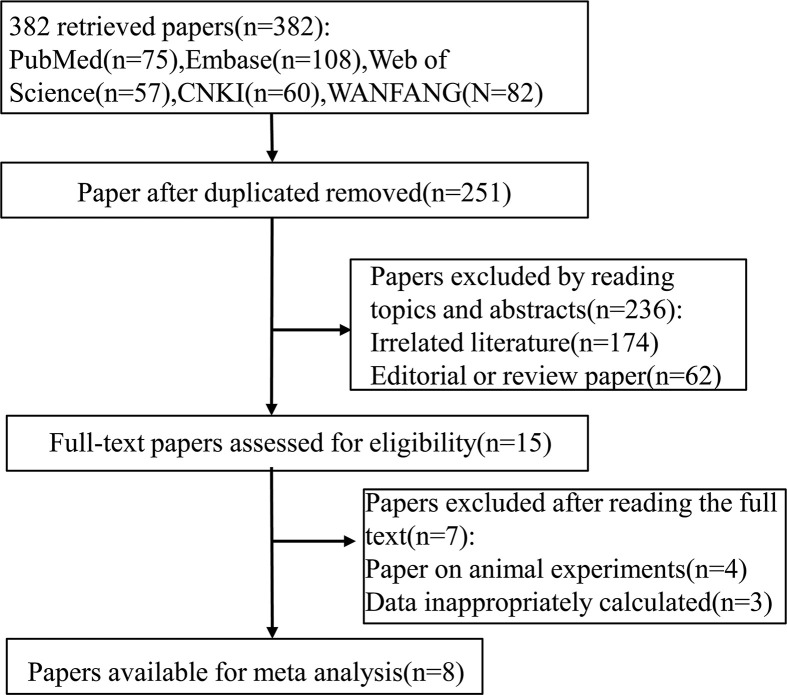
Flow chart of studies included in the meta-analysis

We obtained 382 articles by literature search from the PubMed, Embase, Web of Science, Wanfang, CNKI, and finally 251 articles were kept after eliminating 131 duplicate articles. By reading titles and abstracts, we excluded 236 articles that were not relevant to the study. The full text of the remaining 15 articles were then carefully reviewed. Of the 15 articles, 4 animal experiment studies and 3 with incomplete data or incalculable data after contacting the authors to try get original data were excluded. Therefore, eight articles [7,13–19] satisfying the inclusion criteria were presented in this meta-analysis. The specific retrieval process is shown in Figure 1. Among the eight articles included in the present study, seven articles [7,13–18] compared the expression of miR-145 in breast cancer tissue with that in paracancerous tissue or normal breast tissue, three articles [7,18,19] analyzed the relationship between miR-145 expression and histopathology, and two articles [13,19] discussed the relationship between miR-145 expression and clinical prognosis. The characteristics of the literature included in this meta-analysis are shown in Table 1.

**Table 1:** Characteristics of studies included in this meta-analysis

Reference	Year	Region	Tissue sample and size (case/control)	Quantitative method	Language
Han et al. [7]	2017	China	Carcinoma tissue/breast tissue of healthy women (99/21)	qRT-PCR	English
Quan et al. [13]	2018	China	Carcinoma tissue/paracancerous normal tissues (372/372)	qRT-PCR	English
Liu et al. [14]	2016	China	Carcinoma tissue/paracancerous normal tissues(88/88)	qRT-PCR	English
Zheng et al. [15]	2015	China	Carcinoma tissue/paracancerous normal tissues (39/39)	qRT-PCR	Chinese
Thakur et al. [16]	2016	India	Carcinoma tissue/paracancerous normal tissues (85/85)	qRT-PCR	English
Iorio et al. [17]	2005	Italy	Carcinoma tissue/breast tissue of healthy women (76/10)	Microarray	English
Wang et al. [18]	2012	China	Carcinoma tissue/paracancerous normal tissues (41/41)	qRT-PCR	English
Liu-H et al. [19]	2016	China	Carcinoma tissue/carcinoma tissue (140/117)	qRT-PCR	English

### Meta-analysis results

**Figure 2:**
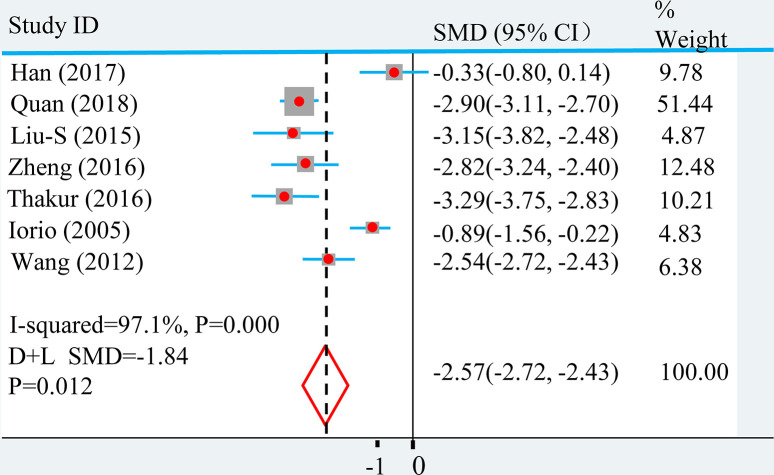
Random-effects SMD for the association of miR-145 expression level and breast cancer The central location and size of the square represent the study-specific SMD and study weight, respectively, and the horizontal lines represent the study-specific 95% CI.

Eight eligible studies analyzed including 1057 breast cancer tissue samples and 656 paracancer or normal breast tissue samples. Among them, the expression of miR-145 in 800 breast cancer tissue samples was compared with 656 paracancerous and normal breast tissue samples. In the process of analysis, we found that there was significant heterogeneity between studies (*I^2^ = 95.5%*; *P*<*0.01*). Pooled SMD for expression of miR-145 comparing breast cancer tissue with paracancerous and normal breast tissue was −2.57 (*95% CI = −2.72 to 2.43*) with a D–L random-effects model, as shown in Figure 2.

**Figure 3:**
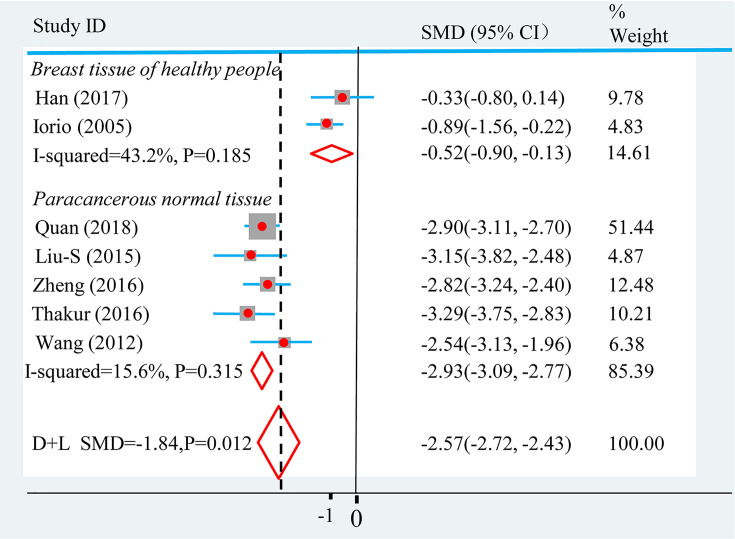
Random-effects SMD for the association of miR-145 expression level and breast cancer The central location and size of the square represent the study-specific SMD and study weight, respectively, and the horizontal lines correspond to the study-specific 95% CI.

To explore the sources of heterogeneity, we conducted sensitivity analysis and found that Han et al.’s study [7] and Iorio et al.’s [17] were the sources of heterogeneity. Re-reviewing these two literatures carefully, we found that the normal breast tissue in the control group of Han et al.’s study [7] and Iorio et al.’s [17] originated from healthy women, while the tissue samples in the other study control groups were paracancerous normal tissues. The tissue source of the control group might be the cause of the heterogeneity of articles, which was confirmed by meta-regression analysis (*P*<*0.001*). Therefore, we conducted the subgroup analysis based on the sample tissue source of the control group, and the internal heterogeneity of the subgroup was significantly reduced (*I^2^* < *50%, P*>*0.10*), as shown in Figure 3. The subgroup analysis showed that miR-145 expression was significantly lower in breast cancer tissues than in adjacent normal tissues (*SMD = −2.93, 95% CI = −3.09* to *−2.77, P<0.0001*), and the expression of miR-145 in breast cancer was significantly lower than that in healthy women (*SMD = −0.52, 95% CI = −0.90* to −*0.13, P<0.01*).

The expression of miR-145 in ER-positive breast cancer patients was significantly lower than that in ER-negative patients (*SMD* = *0.65, 95% CI = 0.378*–*0.916, P<0.001*). There was no difference in the expression of miR-145 between PR-positive breast cancer patients and PR-negative breast cancer patients (*SMD = 0.17, 95% CI =* −*0.10* to *0.44, P=0.920*). Compared with HER-2 negative patients, the expression of miR-145 was significantly lower in HER-2 positive patients (*SMD =* −*1.04, 95% CI =* −*1.38* to −*0.70, P<0.001*). The expression of miR-145 was significantly lower in breast cancer patients with lymph node metastasis than in patients without lymph node metastasis (*SMD =* −*1.75, 95% CI = −2.12* to −*1.38, P<0.001*). Patients with tumors larger than 2 cm in diameter had significantly lower expression of miR-145 than those with tumors less than 2 cm in diameter (*SMD = −1.97, 95% CI = −2.38 to −1.56, P<0.001*). Heterogeneity detection, calculation model and pooled SMD of subgroup analysis are shown in Table 2. In addition, Liu et al. and Thakur et al. analyzed the relationship between the expression level of miR-145 in tumor tissues and tumor tissue grade [16,19]. Both studies suggested that a negative correlation between miR-145 expression and histological tumor grade. However, it was difficult to combine the data with meta-analysis, because there was only an overall comparison in miR-145 expression between grade 3 and grade 1/2 in study by Thakur et al. [16].

**Table 2:** Analysis of miR-145 expression level and different subtype breast cancer

Comparisons	Number of studies	Heterogeneity		Model*	Effect size	
		*I^2^* (%)	*P(Q)*		SMD	*P(Z)*
ER+ vs ER−	3 [7,18,19]	95.4	<0.001	D–L	0.65	<0.001
PR+ vs PR−	3 [7,18,19]	0.0	=0.920	M–H	0.17	=0.223
HER2+ vs HER2−	2 [7,19]	97.7	<0.001	D–L	−1.04	<0.001
LNM+vs LNM−	2 [18,19]	0.0	=0.340	M–H	−1.75	<0.001
TS < 2 vs 2 < TS < 5	2 [18,19]	0.0	=0.787	M–H	−1.97	<0.001

Abbreviations: D–L, Dersimonian–Laird random-effects model; LNM, lymph node metastasis; M–H, Mantel–Haenszel fixed-effects model; *P*(*Q*), *P*-value of *Q* test for heterogeneity; *P*(*Z*), *P*-value of *Z* test for significance test.

### Evaluation of publication bias

**Figure 4:**
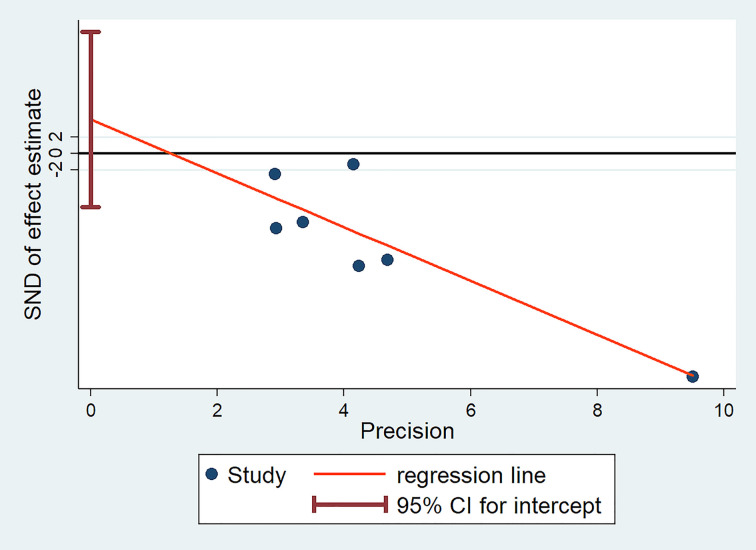
Detection of publication bias

We evaluated the publication bias of the literature included in meta-analysis by Egger’s and Begg’s tests. Egger test is shown in Figure 4. The *P*-value of Egger’s test is 0.366, and that of Begg’s test is 0.230. The results of Egger’s and Begg’s tests showed that the literature included in the meta-analysis had no publication bias.

## Discussion

Of the complicated mechanisms in human cancer, the aberrant expression of miRNA is an important factor. It has been shown that miRNA plays an important role in the occurrence, development, migration and invasion of tumors [20–22]. The expression of miRNA can be accurately detected in fresh and formalin-fixed tissues by qRT-PCR [23]. Therefore, miRNA is believed to be useful for early diagnosis and accurate prognosis, and is expected to become a target of clinical treatment. In recent years, the difference in expression of miR-145 between carcinoma tissues and normal tissues has prompted great interests. Most studies have shown that the expression of miR-145 in breast cancer tissue and serum was decreased, and that the low expression of miR-145 is related to lymph node metastasis and tumor size, which is usually a poor prognostic factor for breast cancer [24–26]. However, recent studies have shown that there is no difference in the expression of miR-145 between breast cancer and normal breast tissue [7].

In view of whether miR-145 expression in breast cancer tissue is down-regulated compared with that in normal breast tissue and paracancerous tissue, it is still controversial. Therefore, in this meta-analysis, we tried our best to search all the papers on the relationship between miRNA expression and breast cancer, and screened the literature on the relationship between miR-145 expression and breast cancer pathology in accordance with the inclusion criteria. We used Egger’s and Begg’s tests to evaluate the publication bias of the included literature. The results showed that the included literature had no publication bias. When analyzing the homogeneity of the literature that have been included in this meta-analysis, we found heterogeneity between the studies. Under the conditions, we selected a D–L random-effects model to calculate the combined SMD and applied a sensitivity analysis method to determine the source of heterogeneity. Then, according to the articles of heterogeneity source, we used the meta-regression analysis method to determine the factors of articles with heterogeneity.

According to sample types in the control, we carried out the subgroup analysis, and the results showed that the expression of miR-145 in breast cancer tissues was significantly lower than that in adjacent tissues and normal breast tissues, which was consistent with the results of most studies [24–27]. In a systematic review by Adhami et al., miR-145 was the most consistently down-regulated miRNA, found in four studies [28]. Our meta-analysis confirmed it. In addition, our study showed that there was no difference in miR-145 expression between PR positive and negative breast cancer patients. The expression of miR-145 in ER positive and HER-2 positive breast cancer patients was significantly lower than that in negative patients, but there were few studies with heterogeneity, the results need to be further confirmed. The expression of miR-145 in breast cancer patients with lymph node metastasis and tumor diameter larger than 2 cm was significantly lower than that in those without lymph node metastasis and tumor diameter smaller than 2 cm, respectively.

Although our meta-analysis represents a quantitative synthesis of all available studies, some limitations of the present study are noted. First of all, the meta-analysis is based on case–control study, and there may have selection bias; second, the analysis of the relationship between miR-145 and specific clinicopathological characteristics of breast cancer, such as ER, PR, HER-2, lymph node metastasis and tumor size etc., due to the relatively small number of studies included, the results need to be further confirmed.

Overall, we have combined the results of all available studies to show that the expression of miR-145 in breast cancer tissue was significantly lower than that in paracancerous tissue and normal breast tissue. Down-regulation of miR-145 expression was not associated with PR status in breast cancer, but was lowly expressed in breast cancer patients with ER-positive, HER-2-positive and lymph node metastasis, and was closely related to tumor size. Independent studies with relatively large sample size based clinical investigations should be conducted before using miR-145 as a diagnostic and prognostic biomarker in breast cancer.

## Abbreviations

CNKI: china national knowledge infrastructure
D–L: Dersimonian–Laird
ER: estrogen receptor
miRNA: microRNA
GRADE: grading of recommendations assessment,development and evaluation
PR: progestogen receptor
qRT-PCR: quantitative real-time polymerase chain reaction
SMD: standardized mean difference
VEGF: vascular endothelial growth factor
95% CI: 95% confidence interval
